# An Insect-Scale Untethered Piezoelectric Robot with Multiple Biomimetic Features

**DOI:** 10.34133/cbsystems.0525

**Published:** 2026-07-23

**Authors:** Jing Li, Zhengxu Yan, Baoyi Liu, Shijing Zhang, Yu Gao, Hongwei Guo, Yingxiang Liu

**Affiliations:** State Key Laboratory of Robotics and Systems, Harbin Institute of Technology, Harbin 150000, China.

## Abstract

Load capacity and speed are 2 essential dimensions in the practical application of miniature robots. In recent years, numerous miniature robots with large load capacity or high speed have been developed. However, it is still a challenge to achieve high speed under large load. Inspired by mythological creatures such as dragon, qilin, and chimera that integrate the characteristics of different animals, an insect-scale tripedal piezoelectric robot incorporating multiple biomimetic features is proposed. By emulating the terrestrial flapping of fish tail, a single driving leg with 2 orthogonal bending vibrations is designed to achieve rapid and flexible motion. The tethered robot is 38 mm in length and weighs 8.6 g, exhibiting a maximum forward speed of 313.49 mm/s (8.25 body length per second), a maximum angular speed of 11.54 rad/s, and a minimum curvature radius of 12.64 mm. By emulating the functional roles of the forelimbs and hindlimbs in otariids galloping, a support scheme combining 2 passive wheels with a driving leg is proposed to realize high speed under large load. The forward speed achieves more than 300 mm/s under 200 g (23.26 times self-weight). Moreover, an untethered robot is fabricated. It exhibits a cost of transport of only 1.91 and can operate continuously for 70 min. The untethered robot demonstrates marked potential for operation in narrow spaces, owing to its small size, superior load characteristics, and high flexibility. We believe that this design method of integrating multiple bionic features can offer a new perspective for enhancing the performance of miniature robots.

## Introduction

Load capacity and speed are 2 essential dimensions in the practical application of miniature robots [1–7]. In recent years, numerous miniature robots with large load capacity or high speed have been proposed [8–14]. However, it remains a challenge for miniature robots to achieve high speed under large load. Traditional miniature robots driven by electromagnetic methods can typically exhibit high speed and simple control [15–18], but the load capacity is limited. For example, a quadruped crawling robot designed by Su et al. [19] reaches a maximum speed of 206 mm/s in the unloaded condition. Nevertheless, its maximum load capacity is only 110 g (1.78 times self-weight), and the speed under this condition is close to zero.

With advances in material technology, shape memory alloys, artificial muscles, pneumatic actuators, piezoelectric actuators, and so on have been developed to drive miniature robots [20–24]. Due to the cooling time required in each actuation cycle, the robots driven by shape memory alloys and artificial muscles are difficult to achieve fast speed [25–32]. Pneumatic actuators operate by deforming a confined space made of flexible materials through pressurized air; the actuation principle also restricts the speed of robots [33–38]. Piezoelectric robots have the advantages of fast response, high power density, and large load capacity, making them a prominent research topic for miniature robots in recent years [39–43]. The miniature piezoelectric robots (MPRs) typically exhibit high speed and large load capacity [44–51]. Under a large load, however, the speed of most existing MPRs drops markedly compared to the maximum. For instance, Li et al. [52] designed an untethered tripedal piezoelectric robot that achieves a maximum speed of 146 mm/s and has a load capacity of 331.6 g (33.8 times self-weight). Under a load of 331.6 g, its speed drops to 62.9 mm/s (43.1% of the maximum). On the other hand, for MPRs, achieving flexible motion typically necessitates multiple DOFs (degrees of freedom). This requirement leads to an increase in the number of actuators, which in turn complicates the control system and structure of the robots.

To achieve high speed under large load and minimize the number of actuators required for multi-DOF motion, we design an insect-scale tripedal MPR. Observations show that fish can achieve flexible terrestrial movement by using their tails to flap at the ground, and otariids can gallop at a high speed on land despite their large body weight [53]. By emulating the 2 features, our proposed miniature robot performs both forward and rotational motion using only one driving leg and achieves high speed under large load. The highlights of this work are detailed below:

1. A design method integrating multiple bionic features is proposed, and a tethered robot with a length of 38 mm and a weight of 8.6 g is developed using this method.

2. Inspired by the terrestrial movement of fish tail, the robot achieves rapid and flexible motion through 2 first-order bending vibrations of a single driving leg in orthogonal direction. It reaches a maximum speed of 313.49 mm/s [8.25 body length per second (BL/s)], a maximum angular speed of 11.54 rad/s, and a minimum curvature radius of 12.64 mm.

3. Inspired by the functional roles of forelimbs and hindlimbs in otariids galloping, the robot achieves high speed under large load through a support scheme comprising 2 passive wheels and a driving leg. Under a load of 200 g (23.26 times self-weight), the forward speed still achieves more than 300 mm/s.

4. A control unit is designed to facilitate wireless control. Our untethered robot exhibits a cost of transport (CoT) of only 1.91, an endurance time of 70 min with a 200 mAh battery, and strong flexibility with experiments of navigating through a narrow environment.

## Materials and Methods

### Manufacture of the driving leg

The balancing weight of the driving leg is made of GB/T 45 steel. The base beam and connecting beam are machined as a single piece from aluminum alloy. The foot is made of alumina ceramic (97% Al_2_O_3_), which offers high hardness and excellent wear resistance. The machining tolerance for the main dimensions of the driving leg components is ±0.02 mm. The assembly is completed by bonding the individual components of the driving leg with EA E-120HP [an epoxy adhesive manufactured by Henkel Loctite (China) Co. Ltd.], followed by a 24-h curing period.

### Circuit schematic diagram of the control unit

The circuit schematic diagram of the control unit is shown in Fig. S1A and B. The control unit consists of 2 main parts: the boost circuit and the control circuit. The current output from the battery is initially amplified to 26.7 V by the boost converter chip and subsequently boosted to +80 V via a charge pump. The control circuit is principally constituted by an ESP32 (a microcontroller unit) and 2 operational amplifier chips. The operational amplifier chips are powered by the +80 V voltage source from the boost circuit. ESP32 generates 2 PWM (pulse-width modulation) signals with adjustable frequencies, which are then amplified to 80 V_p-p_ via the operational amplifier chips. By controlling the frequencies of the PWM signals output by ESP32, two 80 V_p-p_ sinusoidal signals can be simulated. The 2 sinusoidal signals are respectively connected to the PZT (lead zirconate titanate) elements of the driving leg, actuating the robot to move. The relationship among the parts of the control unit is shown in Fig. S1C.

### Measurement and characterization of the robot

As shown in Fig. S3, the vibration modes of the driving leg are tested using a scanning laser Doppler vibrometer system, which comprises 3 subsystems: the scanning laser head, the data management system, and the signal generation system. The scanning laser head scans the vibration velocities on specified plane of the test component. The data management system configures the settings of the scanning laser head, processes the acquired data, and sends frequency sweep commands to the signal generation system. The signal generation system includes a vibrometer controller and a power amplifier. The vibrometer controller generates sweeping frequency signals as requested by the data management system, and the power amplifier amplifies the signals to ensure sufficient power for inducing vibration in the test component.

Fig. S4 illustrates the setups for testing the displacements and trajectories of the foot. The driving leg is fixed on the flat pliers and powered by a power supply. Two laser displacement sensors are positioned at a 90° angle to measure the displacement of the foot in the *Z* and *Y* directions, respectively. By combining the data from the 2 sensors, the motion trajectories of the foot in the *YOZ* plane can be approximated. The entire system is mounted on the air floating vibration isolation platform, which effectively filters out external noise and ensures measurement accuracy.

Fig. S5 shows the setups for testing motion characteristics of the robot. The robot moves within a square testing area of 500 mm × 500 mm. The surface material of the testing area is glass, whose surface has a typical roughness of Ra 0.1 μm and a typical hardness of 5.5 (Mohs scale). The high hardness and smooth surface of the glass are favorable for piezoelectric robot with micrometer vibration amplitudes. A camera is positioned above the testing area to capture motions of the robot, and the record videos are saved on the Orange Pi (a single board computer). The captured images are displayed on the monitor. A pattern is affixed on the tag of the robot. Using a deep learning-trained image recognition model, the center of the pattern on the tag is marked with a red dot in real time by the Orange Pi. The location of the red dot’s pixel in each frame is recorded. By calibrating the relationship between actual distance and the pixel coordinates, the moving distance is obtained, and then the moving speed can be calculated.

The setups for measuring the temperature of the untethered robot are shown in Fig. S11. The experimental process is as follows: The untethered robot is continuously working, and the Honor Band 9 is used for timing. The temperature is recorded at 5-min intervals using a thermal imager. The model of the thermal imager is UTI380 (made by UNI-T, China). The figures are plotted by Origin, Microsoft Office PowerPoint, Adobe Photoshop, and Adobe Illustrator, and the movies are produced by Adobe Premiere Pro.

## Results

### Structure of the miniature robot

**Fig. 1.**
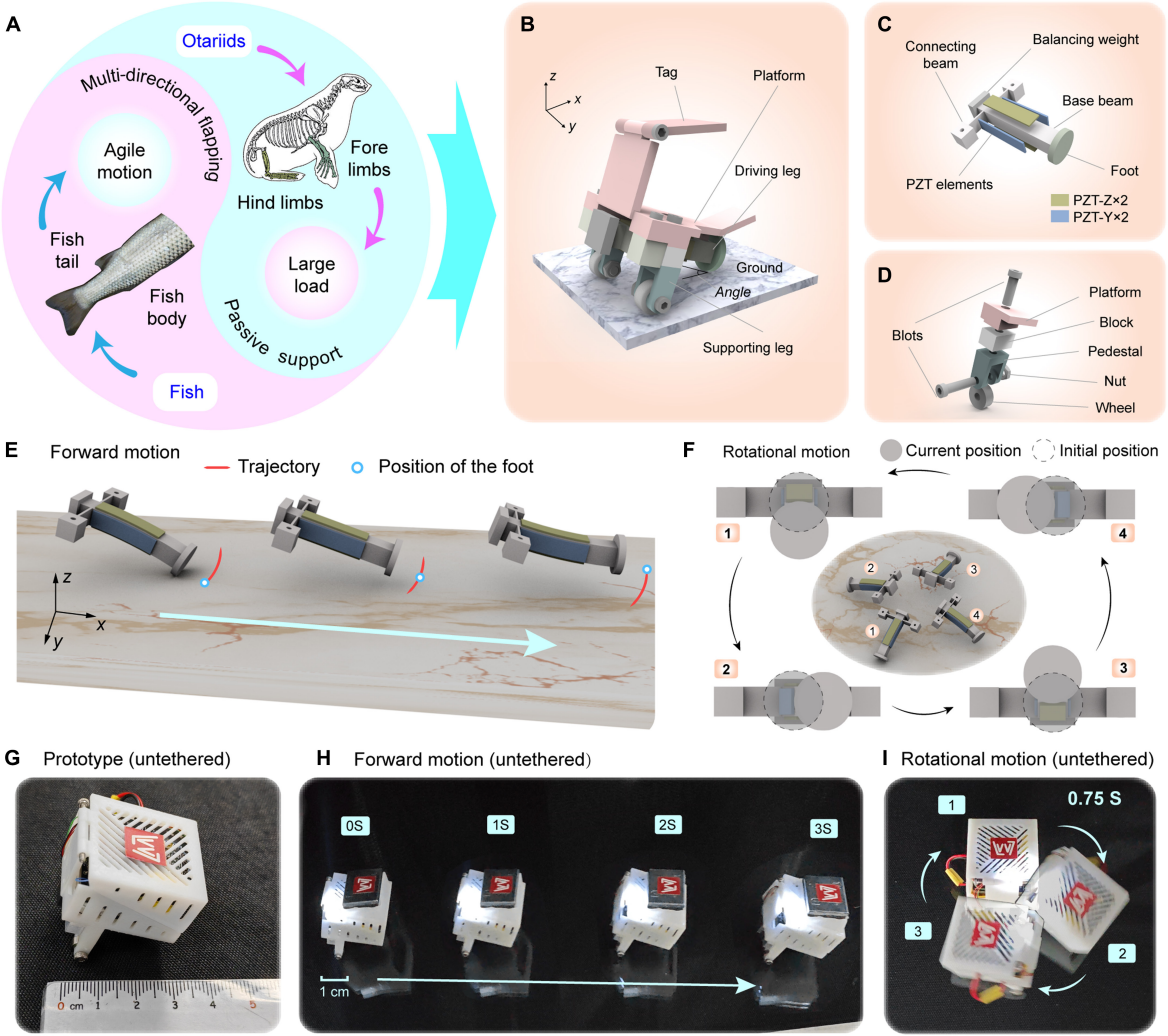
Biomimetic features, detailed structure, actuating principle, and motion characteristic testing of the robot. (A) Biomimetic features of the robot. (B) Configuration of the robot. (C) Main components of the driving leg. (D) Main components of the supporting leg. (E) Actuating principle of forward motion. (F) Actuating principle of rotational motion. (G) Prototype of the untethered robot. (H) Forward motion testing of the untethered robot. (I) Rotational motion testing of the untethered robot.

Mythological images such as the dragon and qilin in Chinese culture and the chimera in ancient Greek mythology are composite entities that amalgamate the traits of different animals. By integrating the characteristics of various animals, they are endowed with powerful capabilities. This idea offers an innovative approach to the design of our robots. Fig. 1A shows the biomimetic features of the robot. We observe that fish can move flexibly on land by flapping their tails against the ground, and otariids can gallop at a high speed with their large body weight by using hindlimbs for propulsion and forelimbs for support. Inspired by these 2 features, a tripedal MPR is proposed. As shown in Fig. 1B and Movie S1, 2 supporting legs and one driving leg are connected to the platform. The tag is on top of the platform. The tethered robot is 38 mm in length and weighs only 8.6 g. The main components of the driving leg are shown in Fig. 1C. The driving leg consists of a balancing weight, 2 connecting beams, a base beam, a foot, and 4 PZT elements. The balancing weight is utilized to adjust the modal characteristics of the driving leg to obtain an ideal vibration mode. The connecting beams are employed to connect the driving leg to the platform and to avoid the vibration coupling with the driving leg. The base beam features a square cross-section, and the 4 piezoelectric elements are attached to its sides. The PZT elements are divided into 2 groups based on their functions: PZT-Z (yellow blocks) and PZT-Y (blue blocks), respectively.

Main components of the supporting leg are depicted in Fig. 1D, where the passive wheel is attached to the pedestal using bolt and nut. The block is between the pedestal and the platform. The angle between the driving leg and the ground (hereinafter referred to as the *Angle*) can be adjusted by varying the thickness of the block. The pedestal, the block, and the platform are fixed together through the upper bolt. As shown in Fig. 1E, forward motion of the robot is generated by the bending vibration in the *ZOX* plane of the driving leg. As depicted in Fig. 1F, rotational motion is achieved through the cooperation between 2 orthogonal bending vibrations of the driving leg, which enables both clockwise and counterclockwise rotation. An untethered prototype is fabricated by integrating a 100 mAh battery and a control unit, as shown in Fig. 1G. It has a body length of 39 mm and weighs 20.9 g. The circuit schematic diagram of the control unit is shown in Fig. S1. It can provide the robot with 2 excitation signals of arbitrary frequency and 0 to 80 V_p-p_ adjustable amplitude. Fig. 1H and I shows the motion characteristic testing of the untethered robot.

### Working principle

**Fig. 2.**
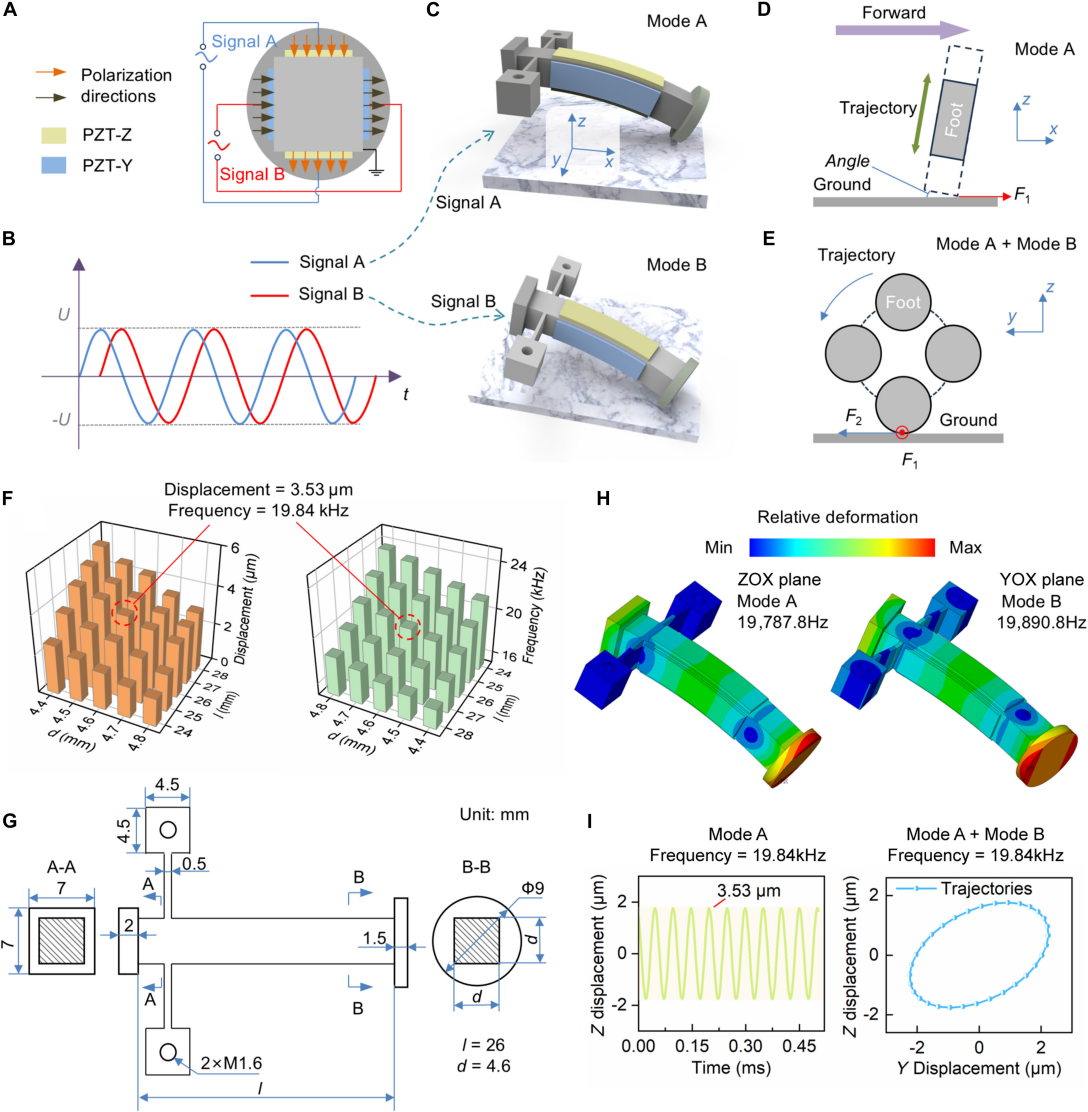
Working principle of the robot and structure parameter determination of the driving leg. (A) Polarization directions of the PZT elements and the wiring of the driving leg. (B) Excitation signals applied to the PZT elements. (C) Different modes of the driving leg. (D) Trajectory of the foot during forward motion. (E) Trajectory of the foot during rotational motion. (F) Simulation results of the maximum displacements of the foot in the *Z* direction and the average resonance frequencies of the first-order bending vibration of the driving leg under different values of *d* and *l*. (G) Structure parameters of the driving leg. (H) Simulation results of first-order bending vibration modes of the driving leg in the *ZOX* and *YOX* planes. (I) Simulated displacements of the foot in the *Z* direction and trajectories of the foot.

Fig. 2A shows polarization directions of the PZT elements and the wiring of the driving leg. Viewed from the square cross-section of the driving leg, the polarization directions of PZT elements on opposite sides are the same. The excitation signals for PZT-Z and PZT-Y are signal A and signal B, respectively, and the base beam of the driving leg is connected to the signal ground. As shown in Fig. 2B, both signal A and signal B are sinusoidal, with a phase difference of 90° (a quarter period). As depicted in Fig. 2C, when signal A is applied independently, one PZT element of PZT-Z stretches, while the other shortens under the action of electric field, resulting in the bending vibration of the driving leg in the *ZOX* plane. Similarly, when signal B is applied independently, the driving leg generates bending vibration in the *YOX* plane. The above modes are designated as mode A and mode B, respectively. The deformation principle of the driving leg is shown in Fig. S2 and Note S1. As shown in Fig. 2D and Movie S1, when mode A acts alone, the bending vibration of the driving leg in the ZOX plane induces a tendency for the foot to move backward relative to the ground, which in turn generates a reaction force *F*_1_ from the ground in the positive *X* direction, driving the robot forward. When mode A and mode B act together, as shown in Fig. 2E and Movie S1, the bending vibrations in the *ZOX* and *YOX* planes of the driving leg produce a counterclockwise elliptical trajectory at the foot, due to the 90° phase difference between the 2 signals. In this situation, the reaction force between the foot and the ground can be decomposed into *F*_1_ along the positive *X* direction (the same as *F*_1_ in Fig. 2D) and *F*_2_ along the positive *Y* direction. The component *F*_1_ impedes rotational motion, leading to an increased curvature radius. The component *F*_2_ induces a clockwise rotational motion. Similarly, if the phase difference between the 2 excitation signals is 270° (3 quarters period), the rotational motion direction will change to counterclockwise. Utilizing only one driving leg, the robot is capable of both forward and rotational motions.

### Structure parameter determination

In this section, the following goals are achieved by adjusting *d* and *l*, the side length for cross-section and the length of the base beam:

1. The audible range of human hearing is approximately 20 Hz to 20 kHz. To minimize operational noise while maximizing the displacement of the foot (lower resonant frequency generally yields larger amplitude) and accounting for hardware limitations (high resonant frequency exceeding the circuit’s switching capability causes waveform distortion), the average resonance frequency of the first-order bending vibrations in the *ZOX* and *YOX* planes of the driving leg should therefore be close to 20 kHz.

2. To ensure the robot’s mobility on a plane with high surface roughness, the maximum simulated displacement of the foot in the *Z* direction should be at least 3.2 μm under a voltage of 10 V_p-p_. This threshold corresponds to a surface roughness of Ra 3.2 μm—a medium-to-coarse finish typical for general precision requirements.

Fig. 2F shows the simulation results of the maximum displacements of the foot in the Z direction and the average resonance frequencies of the driving leg under different values of *d* and *l*. By comparing the conditions mentioned above with the simulation results, the values of *d* and *l* are chosen as 4.6 and 26 mm, respectively. Under this condition, the average resonance frequency of the driving leg is 19.84 kHz, and the maximum displacement of the foot in the *Z* direction is 3.53 μm, both of which satisfy the requirements. The remaining parameters are determined by comprehensively considering the manufacturing complexity and compactness of structure, as shown in Fig. 2G. The modal analysis results in the *ZOX* and *YOX* planes of the driving leg under these parameters are presented in Fig. 2H. The method for achieving mode degeneration in the *ZOX* and *YOX* planes is described in Note S2. The displacements of the foot in the *Z* direction and trajectories of the foot, simulated under 10 V_p-p_ excitation signals, are depicted in Fig. 2I.

### Experiments with external power supply

#### Test of vibration modes and trajectories

**Fig. 3.**
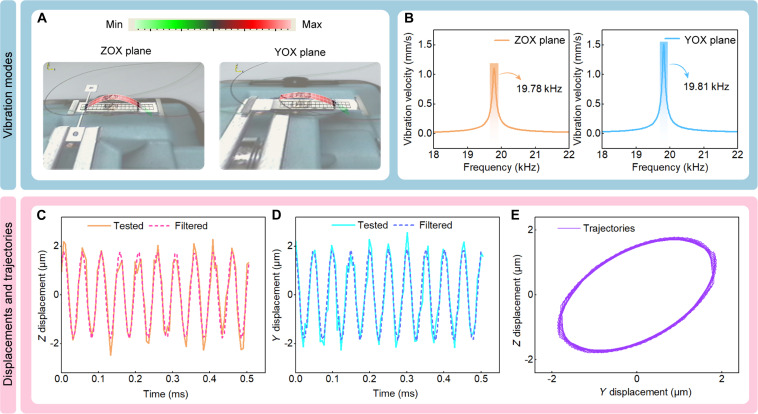
The vibration mode testing of the driving leg and trajectory testing of the foot. (A) Vibration shapes of the driving leg in the *ZOX* and *YOX* planes. (B) Vibration velocity response spectrums of the first-order bending vibrations in the *ZOX* and *YOX* planes. (C) Tested and filtered data for the displacement in the *Z* direction. (D) Tested and filtered data for the displacement in the *Y* direction. (E) Trajectories in the *YOZ* plane.

As shown in Fig. S3, the vibration modes of the driving leg are tested using a scanning laser Doppler vibrometer system. The measured results of the modes in the *ZOX* and *YOX* planes are presented in Fig. 3A and B. The driving leg exhibits first-order bending vibration in the *ZOX* and *YOX* planes, with resonant frequencies of 19.78 and 19.81 kHz, respectively. The difference between them is only 0.03 kHz, which shows the success of mode degeneration.

Fig. S4 illustrates the setups for testing the displacements and trajectories of the foot. Signal A and signal B are used to excite PZT-Z and PZT-Y (as shown in Fig. 2A and B). The phase difference between the signals is 90°, the frequencies are both 19.80 kHz (based on the results in Fig. 3B), and the voltages are both 10 V_p-p_. Fig. 3C and D illustrates the displacement data for 10 cycles in the *Z* and *Y* directions of the foot. From the filtered data, the displacements in the *Z* and *Y* direction are both about 4.0 μm. As shown in Fig. 3E, the trajectories of the foot are elliptical in shape, verifying the feasibility of generating the expected elliptical vibration trajectory in Fig. 2E.

#### Motion characteristic testing

**Fig. 4.**
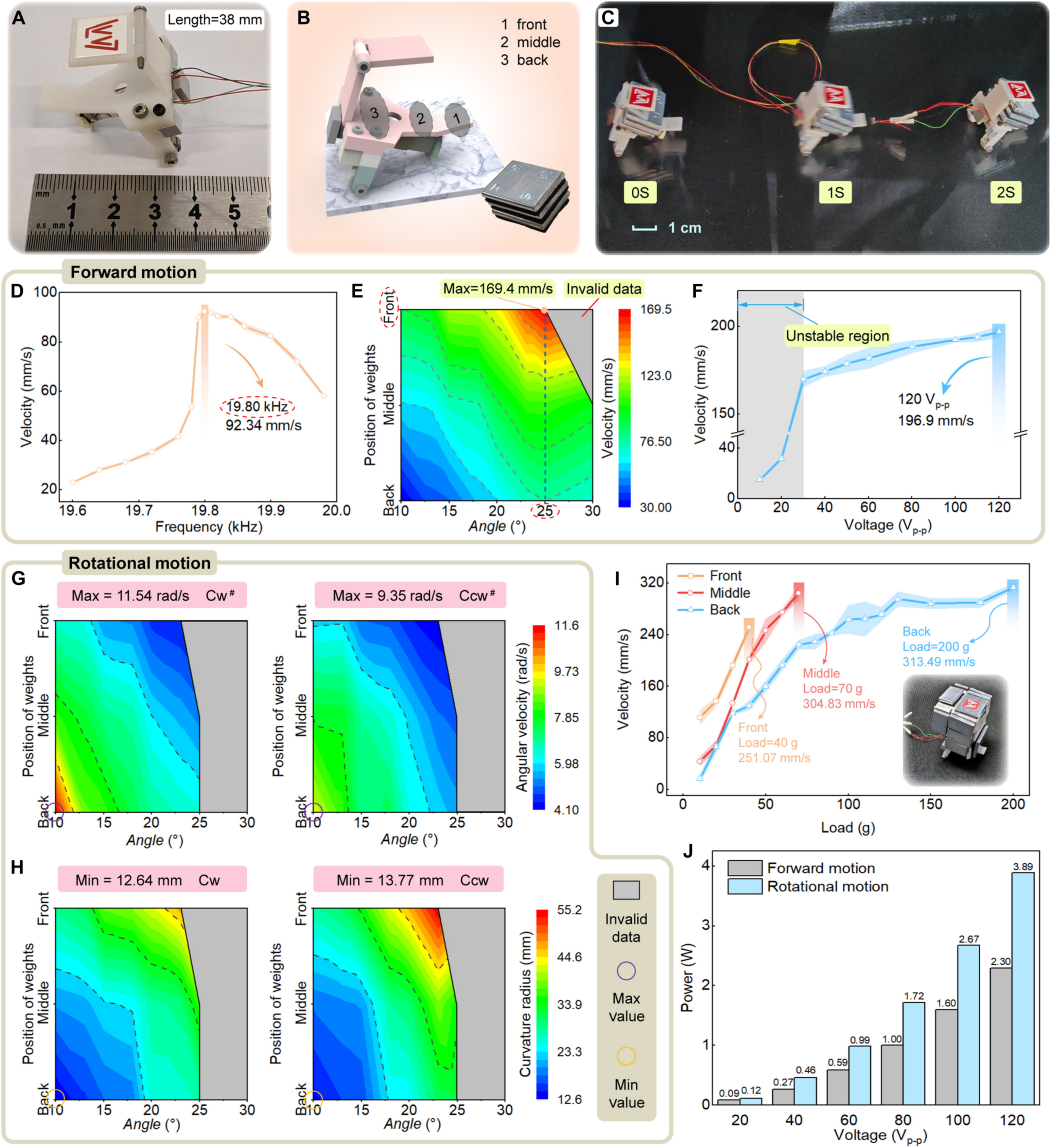
Forward motion testing, rotational motion testing, load characteristic, and power consumptions of the robot. (A) Prototype of the tethered robot. (B) Position of weights put on the robot. (C) Display of the forward motion process. (D) Forward velocity versus signal frequency. (E) Forward velocity versus *Angle* and position of weights. (F) Forward velocity versus exciting voltage. (G) Angular velocity for clockwise and counterclockwise rotations versus *Angle* and position of weights. (H) Curvature radius versus *Angle* and position of weights. (I) Load characteristic testing. (J) Power consumptions of forward and rotational motions under various exciting voltages. # Cw and Ccw denote clockwise and counterclockwise, respectively. The same applies below. Note: The background shading in (D), (F), and (I) represents error bands.

The setups for testing motion characteristics of the robot are shown in Fig. S5. First, forward experiments are carried out to find the maximum speed. The effects of the signal, the *Angle*, and the position of weights are examined. Eight angles are selected: 10°, 13°, 15°, 18°, 20°, 23°, 25°, and 30°. The *Angle* is adjusted by varying the thickness of the block (as shown in Fig. 1D and Fig. S6). The *Angle* is 10° when no block is used. The tethered prototype is shown in Fig. 4A, with a body length of 38 mm and weighing 8.6 g. Due to the light self-weight, the pressure exerted by the foot on the ground is insufficient, leading to poor motion performance; thus, a weight of 30 g is placed on the robot’s platform to address the problem. As shown in Fig. 4B, the positions of weights at 1, 2, and 3 are designated as front, middle, and back, respectively. A display of the forward motion process for tethered prototype is shown in Fig. 4C. As shown in Fig. 4D, the velocity versus the frequency of the signal is studied, firstly, with the exciting voltage at 30 V_p-p_, the *Angle* at 20°, and the position of weights at middle. The forward speed reaches a maximum of 92.34 mm/s (2.43 BL/s) at the frequency of 19.80 kHz. Then, the velocity versus the *Angle* and the positions of weights is studied at a frequency of 19.80 kHz and a voltage of 30 V_p-p_. As shown in Fig. 4E and Movie S2, with other conditions constant, the forward speed increases as the weights are positioned closer to the front and the *Angle* is greater. On the one hand, the phenomenon can be attributed to the fact that increasing the *Angle* and positioning the weight closer to the front increases the pressure exerted by the foot on the ground, thereby reducing relative sliding. On the other hand, as the *Angle* increases, the component for the displacement of the foot along the *X* direction increases (as shown in Fig. 2D), meaning that the deformation driving the robot forward is enhanced. When the *Angle* is 30° and the position of weights is front, the robot loses stability and fails to obtain reliable data. The maximum forward speed is achieved when the *Angle* is 25° and the position of weights is front, with a value of 169.4 mm/s (4.46 BL/s). Finally, with the signal frequency at 19.80 kHz, the *Angle* at 25°, and the position of weights at front, the velocity versus the exciting voltage is studied. As shown in Fig. 4F, the velocity increases as the voltage increases. An obvious increase in speed is observed between 20 and 30 V_p-p_. This is because below 30 V_p-p_ the signal is too weak for the driving leg to vibrate reliably under load, so the motion is unstable. Above 30 V_p-p_, the stronger signal drives effective vibration and speed is significantly increased. When the voltage is 120 V_p-p_, the forward speed reaches the maximum value, which is 196.9 mm/s (5.18 BL/s).

Then, the impact of the *Angle* and the position of weights on rotational motions is examined. The variable values are consistent with forward motion testing. The assessment of rotational motion focuses on 2 indexes: angular velocity and curvature radius. Higher angular velocity and smaller curvature radius correlate with faster reaction speed and better flexibility. As shown in Fig. 4G and H, the maximum angular speed is 11.54 rad/s, and the minimum curvature radius is 12.64 mm. These values are measured when the robot rotates clockwise under the condition that the *Angle* is 10° and the position of weights is back. As shown in Movie S3, with other conditions constant, the angular velocity increases and the curvature radius shrinks as the *Angle* decreases and the weights are positioned closer to the back. This phenomenon arises primarily from the following 2 reasons:

1. When the weights are positioned closer to the back, the moment of inertia *I* during rotation decreases. Meanwhile, as the *Angle* decreases, less energy is required to lift the driving leg, allowing more energy *Ek* to be allocated to rotation. According to the rotational kinetic energy formula [Eq. (1)], the angular velocity *ω* increases.

$$E_{k}=\frac{1}{2}I\omega^{2}$$ (1)

2. During rotation, the projection of the elliptical vibration trajectory at the foot onto the *ZOX* plane is identical to that in forward motion—both are oblique trajectories. As shown in Fig. S7, when the *Angle* decreases, the projection component of the foot vibration trajectory in the positive *X* direction reduces (from Δ*l*_1_ to Δ*l*_2_). Following the previous analysis of rotational motion principles, this projection component impedes rotation and leads to an increased radius of curvature. Consequently, decreasing the *Angle* reduction results in a smaller curvature radius.

The patterns of clockwise and counterclockwise rotation are consistent, but specific data exhibit certain differences due to processing and assembly errors. The gray area depicted in Fig. 4G and H indicates the absence of valid data, as the robot cannot maintain stability in this situation. The display of the rotational motion processes under different conditions is shown in Fig. S8.

#### Load characteristic and power consumption testing

Fig. 4I and Movie S4 investigates the velocity versus the load on the robot under the condition of signal frequency of 19.80 kHz, voltage of 120 V_p-p_, and the *Angle* of 25°. The weights are placed at the front, middle, and back (shown in Fig. 4B), and the load is incrementally increased at each position until the robot loses stability. For a given position, the forward velocity increases with increasing weight. At any fixed mass, the forward velocity increases as the weights are positioned closer to the front. This indicates that (a) Increasing the mass and placing the weights closer to the front raise the pressure exerted on the foot, thereby reducing slippage in the *X* direction; (b) Owing to the driving leg and passive wheel support scheme, increased load mass has a minor influence on motion resistance. A detailed analysis of the above 2 points is provided in Note S3 and Fig. S9.

The 2 factors mentioned above enable the robot to maintain high speed under large load. In the load characteristic experiment, the robot reaches a maximum forward velocity of 313.49 mm/s (8.25 BL/s) under a 200-g load (23.26 times self-weight), exceeding the value obtained in forward motion testing. This indicates that enhancing the contact conditions between the foot and the ground through an appropriate increase in load can lead to a faster speed.

The robot exhibits superior load characteristics. In order to quantify the robot’s motion capability under large load, we introduce a parameter *λ* defined as:

$$\lambda ={\left[\frac{m_{\text{load}}}{m_{\text{robot}}}\right]}^{1+\gamma }\cdot {\left[\frac{V}{v_{0}}\times \frac{V}{L}\right]}^{1-\gamma }$$ (2)

where *m*_load_ is the load mass; *m*_robot_ is the robot’s own mass; *V* and *v*_0_ are the maximum velocities attained under current load and no-load conditions, respectively; and *L* is the body length. *γ* is the importance exponent with a range of (−1, 1), reflecting the relative emphasis of the metric *λ* on load capacity versus maintaining high speed under load. If *γ* > 0, greater weight is assigned to the former. If *γ* < 0, greater weight is assigned to the latter. The value of *γ* can be selected based on the application scenario. In this work, we consider both capabilities equally important, and therefore set *γ* = 0. A larger *λ* indicates a stronger capability for maintaining high speed under large load.

Based on the load characteristic testing results, the *λ* values for our tethered robot are computed for each load mass. Due to the light self-weight, the pressure exerted by the driving leg’s foot on the ground is insufficient under no load conditions, preventing reliable measurement of *v*_0_. We therefore adopted the maximum speed obtained at a 30-g load from the motion characteristic experiment as a surrogate for *v*_0_. This substitution does not compromise fairness, as *v*_0_ appears in the denominator of the formula, and the maximum speed at 30 g is evidently greater than that under no-load conditions. As shown in Fig. S1A, the *λ*_max_ of the tethered robot is 312.8 under a 200-g load.

As shown in Fig. 4J, the power consumptions of the robot in forward and rotational motions are measured. The robot is respectively excited by signals with voltages of 20 V_p-p_, 40 V_p-p_, 60 V_p-p_, 80 V_p-p_, 100 V_p-p_, and 120 V_p-p_ at a frequency of 19.80 kHz. The power consumptions for forward motion are 0.09, 0.27, 0.59, 1.00, 1.60, and 2.30 W, respectively. For rotational motion, the power consumptions are 0.12, 0.46, 0.99, 1.72, 2.67, and 3.89 W, respectively. For piezoelectric robots, power consumption depends solely on excitation voltage and shows no marked correlation with load mass. The flexibility of the tethered robot is assessed through a test, with the testing process depicted in Fig. S11 and Movie S5.

### Experiments of the untethered robot

#### Motion characteristic testing of the untethered robot

**Fig. 5.**
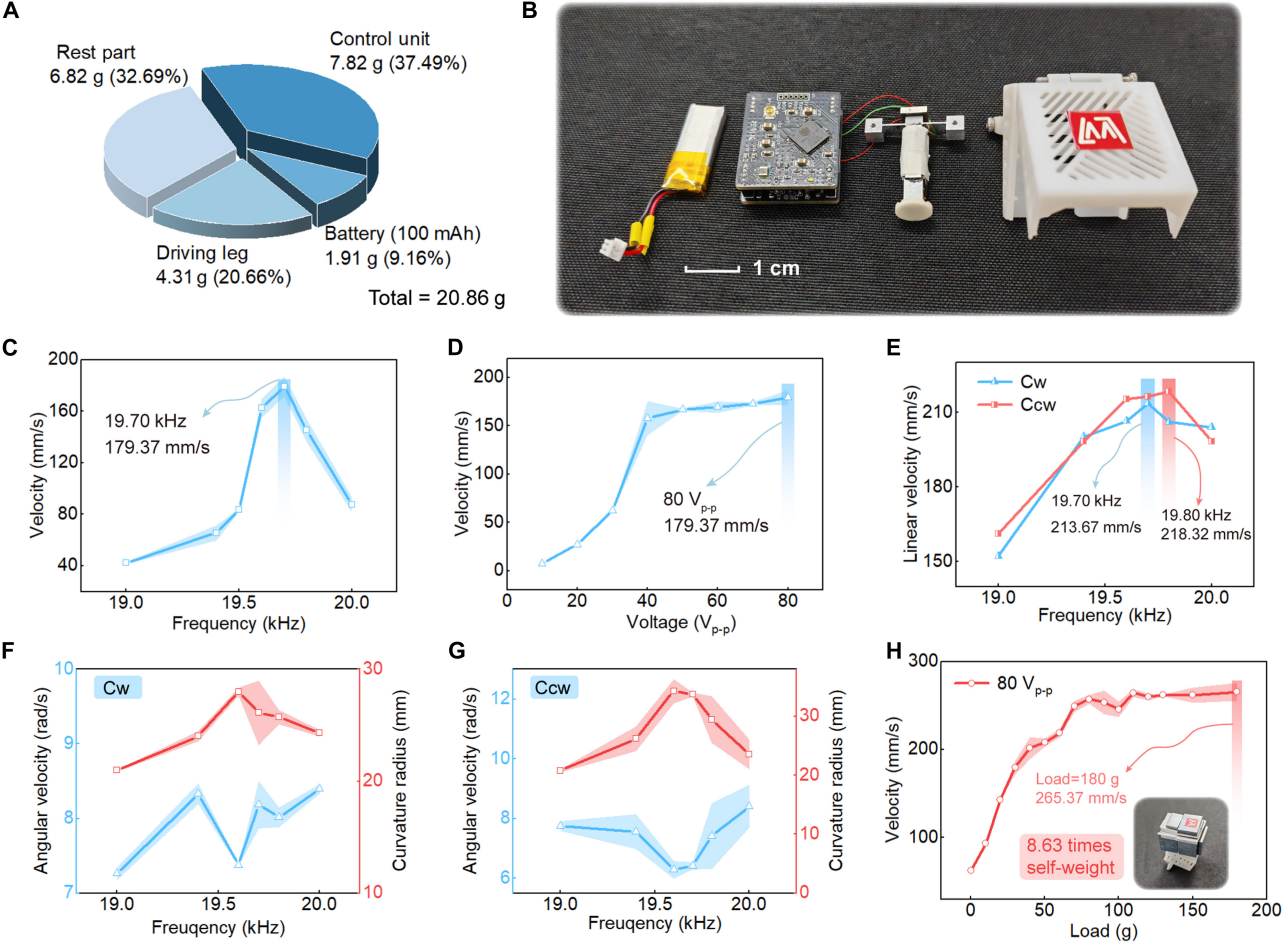
Weight distribution, motion characteristic testing, and load characteristic of the untethered robot. (A) Weight distribution of the untethered robot. (B) Photograph of the individual components. (C) Forward velocity versus signal frequency. (D) Forward velocity versus signal voltage. (E) Linear velocity for clockwise and counterclockwise rotation versus signal frequency. (F) Angular velocity and curvature radius for clockwise rotation versus signal frequency. (G) Angular velocity and curvature radius for counterclockwise rotation versus the signal frequency. (H) Load characteristic testing of the untethered robot. Note: The background shading in (C), (D), and (F) to (H) represents error bands.

As shown in Fig. 5A, the untethered robot has a total weight of 20.86 g, with the control unit weighing 7.82 g, the battery (100 mAh) weighing 1.91 g, the driving leg weighing 4.31 g, and the remaining part weighing 6.82 g. Fig. 5B presents a photograph of the individual components of the untethered prototype. To enhance the contact conditions between the foot and the ground, a 30-g load is added to the robot during forward characteristic testing. Firstly, the forward motion characteristic is evaluated. Fig. 5C shows the forward velocity versus frequency at 80 V_p-p_. The maximum forward velocity reaches 179.37 mm/s (4.60 BL/s) when the frequency is 19.70 kHz. Fig. 5D presents the forward velocity versus excitation voltage at 19.70 kHz. The forward velocity increases monotonically with voltage.

Subsequently, the rotational motion characteristic of the untethered robot is evaluated. Fig. 5E illustrates the relationship between linear velocity and frequency for clockwise and counterclockwise rotation at a voltage of 80 V_p-p_. The maximum linear velocity is 213.67 mm/s at 19.70 kHz for clockwise rotation and 218.32 mm/s at 19.80 kHz for counterclockwise rotation. Fig. 5F and G depicts the variations in angular velocity and curvature radius with frequency for clockwise and counterclockwise rotation, respectively. The displacement component of the foot in the *X* direction reaches its maximum at a frequency of 19.70 kHz during rotational motion (as can be observed from Fig. 5C). As the signal frequency approaches this value, the displacement component that hinders the robot’s rotation increases. Therefore, the curvature radius of the rotational motion increases as the frequency approaches 19.70 kHz. Angular velocity is defined as the ratio of linear velocity to the curvature radius. Since it is influenced by these 2 variables, its variation with signal frequency does not exhibit a clear trend. The motion characteristic testing of the untethered robot is shown in Movie S6.

#### Load characteristic testing of the untethered robot

Load characteristic testing is conducted with the untethered robot, as shown in Fig. 5H and Movie S7. The excitation signal is set to 80 V_p-p_ at a frequency of 19.70 kHz. The maximum forward velocity is 265.37 mm/s (6.80 BL/s) under a load of 180 g (8.63 times self-weight). The results demonstrate that our untethered robot retains the same high-speed performance under large load as the tethered version. The *λ* values for the untethered robot are computed for each load mass, as shown in Fig. S10B. The *λ*_max_ is 249.6 under a load of 180 g.

#### Demonstrative experiments of the untethered robot

**Fig. 6.**
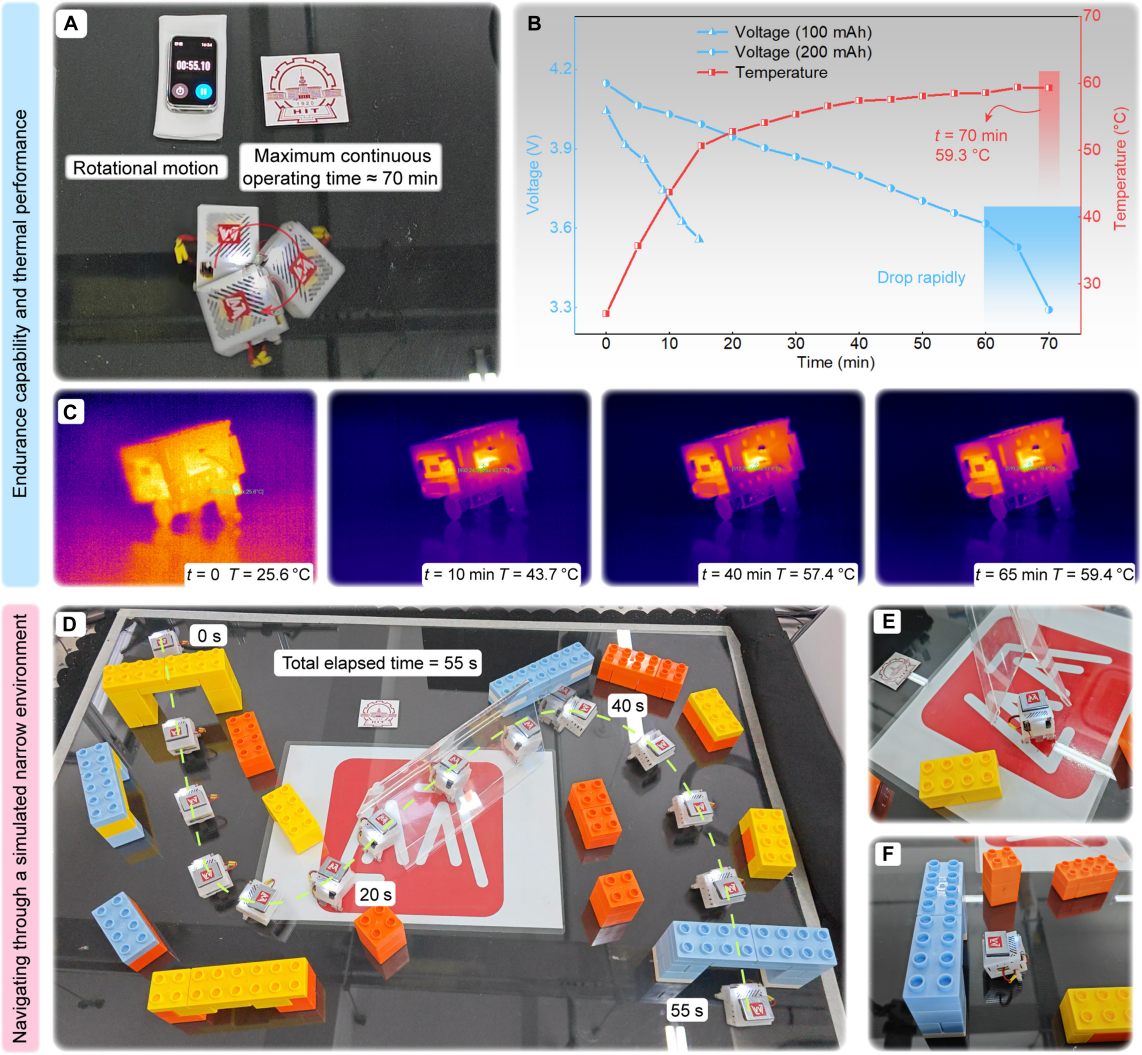
Demonstrative experiments of the untethered robot. (A) Display of the motion process in endurance capability testing. (B) Voltage of batteries and temperature versus time when the untethered robot operates continuously. (C) Thermal images for 0 (initial state), 10 min, 40 min, and 65 min. (D) Display of the motion process in navigating through a simulated narrow environment. (E) Display of the untethered robot through the narrow tunnel. (F) Display of the untethered robot reaching the finish line.

Fig. 6A and Movie S8 show the experimental process for testing the endurance capability of the untethered robot. The untethered robot is continuously rotated counterclockwise until the battery power is exhausted, with the Honor Band 9 used for timing. The excitation voltage is set at 40 V_p-p_. Tests are performed with 100- and 200-mAh batteries. Fig. 6B presents the battery voltage versus time for both capacities. The 100- and 200-mAh batteries can sustain the untethered robot continuous motion for approximately 15 and 70 min, respectively. Due to its higher internal resistance, the 100-mAh battery exhibits a more pronounced voltage drop under discharge compared with 200-mAh battery. Its terminal voltage falls below the 3.3-V threshold prematurely, so residual energy remains unused and unavailable to power the robot. The load characteristic testing indicates that our untethered robot can readily accommodate a battery of even larger capacity, enabling missions of long duration or long distance.

As miniature robots shrink, the surface area of PCB (printed circuit board) for heat dissipation is reduced, and the temperature rise induced by component heating becomes marked. Sustained high temperatures can raise power consumption, cause device failure, and degrade performance. Therefore, a thermal performance experiment is conducted on the untethered robot, and the results are presented in Fig. 6B. The robot starts at 25.6 °C and reaches a maximum temperature of 59.4 °C after 65 min of continuous operation. The temperature rise *Δ_T_* is kept to a moderate 33.8 °C. The structural body material of the robot is “Future 8300 High-Temperature Resin” from Shenzhen Future Factory Additive Technology Co. Ltd. It has a heat deflection temperature of 100 to 105 °C, which is well above the maximum temperature of 59.4 °C. Therefore, there is no concern regarding creep, softening, or other structural strength degradation of the robot’s structural material due to temperature rise during prolonged operation. Thermal images of the robot working continuously for 0 (initial state), 10 min, 40 min, and 65 min are shown in Fig. 6C. The model of the thermal imager is UTI380 (made by UNI-T, China), and the measurement process is shown in Fig. S12.

Fig. 6D and Movie S9 presents the movement trajectory of the robot navigating through a simulated narrow environment. Under manual remote control, the robot successfully navigates through the narrow tunnel and reaches the finish line (as shown in Fig. 6E and F). The total duration of the experimental process is 55 s. This experiment shows that the robot has good flexibility and great potential to work in narrow places.

## Discussion and Conclusion

### Discussion

A comparison of our proposed robot with other similar works is presented in Table 1. Among the comparative robots, our robot achieves multi-DOF (one linear and one rotational) motion with the fewest number of actuators (only one), aligning with the robot proposed by Xun et al. [45]. In terms of load characteristics, our robot also achieves notable performance. With a maximum load of 200 g (23.26 times self-weight), our tethered robot reaches the maximum forward velocity of 313.49 mm/s (8.25 BL/s). It should be noted that the motion performance of piezoelectric robots is highly dependent on the surface condition. All the motion data reported above for our robot is measured on a smooth glass surface. Additionally, we conduct motion experiments on other material surfaces, such as desktop and marble, as shown in Fig. S13. Our robot possesses a certain capability for motion on various surfaces, yet performs better on smooth, hard surfaces such as glass.

**Table 1.** Comparisons with other miniature robots. Data with the symbol “*” are approximately estimated by using the figures and values from the references.

	Our tethered robot	Our untethered robot	Liu et al. [1]	Zhou et. al [12]	Wu et al. [42]	Wei et al. [43]	Xun et al. [45]	Li et al. [52]
DOF	1L+1R	1L+1R	1L+1R	2L+1R	1L	1L+1R	1L+1R	1L
Number of actuators	1	1	6	3	4	4	1	2
Length/mm	38	39	58	55	46	30	56	31
Weight/g	8.6	20.9	42.55	45	1.8	25.2	92.0*	9.8
Max velocity/(mm·s^-1^)/(BL·s^-1^)	313.49/8.25	265.37/6.80	516.3/9	31.5/0.57	486.6/10.6	562/18.7	617.1/11	146.0/4.7
Load/g /Times its weight	200 (23.26 times)	180 (8.63 times)	200 (4.7 times)	200 (4.4 times)	5.5 (3.05 times)	485 (19.25 times)	300 (3.26 times)	331.6 (33.8 times)
Velocity under load_max_ /(mm·s^-1^)/(BL·s^-1^)	313.49/8.25	265.37/6.80	200*/3.4	31.4/0.57	50*/1.1	0*	440.1/7.9	62.9/2.0
*λ*_max_ ^a^	312.8	249.6	10.13*	2.92	5.03*	49.97*	20.45	45.22
CoT	3.59	1.91	36.3	287.9*	-	-	-	5.56

^a^ For every robot in Table 1, the maximum λ value obtained across all tested load is used for comparison.

We calculate the *λ* for the robots in Table 1 and take the maximum values as the reference. The variation of the *λ* with load for the robots is shown in Fig. S14. The *λ*_max_ values of our tethered and untethered robot are 312.8 and 249.6, respectively, which are much larger than these similar miniature robots. This indicates that our robot can achieve high speed under large load.

CoT serves as a key metric for assessing energy consumption in miniature robots. In essence, it represents the energy required to move a unit weight over a unit distance. The lower the CoT value, the higher the robot’s energy efficiency. Based on the data from load characteristic testing and power consumption testing, we calculate the CoT versus load curves for the tethered and untethered robots, as shown in Fig. S15. With a load of 200 g, our tethered robot exhibits a minimum CoT of 3.59. With 180 g, our untethered robot exhibits a minimum CoT of 1.91. As shown in Fig. S16, both our tethered and untethered prototypes achieve CoT values at a low level among the similar MPRs used for comparison. This indicates that our robot achieves high energy utilization efficiency. The detailed calculation process for the minimum CoT of our robot is documented in Note S4.

### Conclusion

Inspired by mythological creatures such as dragon, qilin, and chimera that integrate the characteristics of different animals, an insect-scale MPR imitating the movements of fish tail and otariids is proposed in this paper. On the one hand, a driving leg with orthogonal bending vibrations is designed by imitating the flapping motion of fish tail, which helps the robot achieve flexible forward and rotational motions. The forward and angular speed reach up to 281.5 mm/s (7.4 BL/s) and 11.54 rad/s, respectively. The minimum curvature radius is 12.64 mm. On the other hand, a foot–wheel coordination support scheme is proposed by imitating the functional roles of the forelimbs (support) and hindlimbs (propulsion) in otariids galloping motion on land. It makes the robot achieve a speed of 313.49 mm/s (8.25 BL/s) when the load reaches 200 g (23.26 times self-weight). This performance indicates superior load characteristics. Moreover, an untethered robot is designed by integrating a battery and a control unit. The prototype is 39 mm in length and weighs 20.9 g. It exhibits a CoT of only 1.91 and can operate continuously for 70 min powered by a 200-mAh battery. Our untethered robot demonstrates low power consumption and marked potential for narrow space operations. In future work, efforts will focus on integrating visual sensors for closed-loop control to improve task adaptability.

## Data Availability

All data supporting the findings of this study are available in the paper and the Supplementary Materials.

## Supplementary Materials

Supplementary 1 Notes S1 to S4 Figs. S1 to S16 Movies S1 to S10
