# Supplementary material for: An Insect-Scale Untethered Piezoelectric Robot with Multiple Biomimetic Features
Source: Cyborg Bionic Syst. 2026 Jul 23;7:0525. doi: 10.34133/cbsystems.0525 (PMC13403209; doi:10.34133/cbsystems.0525)
Supplement: Supplementary 1 — Notes S1 to S4 Figs. S1 to S16 Movies S1 to S10 [file cbsystems.0525.f1.zip › 3_Supplementary_Material_0516.docx]

Supporting Information

Title

An Insect-scale Untethered Piezoelectric Robot with Multiple Biomimetic Features

**Authors**

Jing Li†, Zhengxu Yan†, Baoyi Liu†, Shijing Zhang, Yu Gao, Hongwei Guo, Yingxiang Liu*

**Affiliations**

State Key Laboratory of Robotics and System, Harbin Institute of Technology, China

†These authors contributed equally to this work.

*Address correspondence to: liuyingxiang868@hit.edu.cn

**The Supporting Information includes:**

**Note S1** Deformation principle of the driving leg.

**Note S2** The method for achieving mode degeneration in the ZOX and YOX planes.

**Note S3** Analysis of the tethered robot load characteristic testing results.

**Note S4** Detailed calculation process of the CoT of our robot.

**Fig. S1** Circuit schematic diagram of the control unit.

**Fig. S2** Deformation principle of the driving leg.

**Fig. S3** Experimental setups for vibration modes testing of the driving leg.

**Fig. S4** Experimental setups for testing the displacements and trajectories of the foot.

**Fig. S5** Experimental setups for testing motion characteristics of the robot.

**Fig. S6** Method for adjusting the angle between the driving leg and the ground.

**Fig. S7** Influence of the *Angle* variation on the projection component of foot vibration trajectory in the positive X direction.

**Fig. S8** Display of the rotational motion processes of the robot under different conditions.

**Fig. S9** Simplified static force model of the robot under load.

**Fig. S10** Variation of the λ values with load mass for both tethered and untethered robots.

**Fig. S11** Flexibility testing of the robot.

**Fig. S12** Experimental setups for measuring the temperature of the untethered robot.

**Fig. S13** Motion experiments of the robot on cardstock, desktop, glass, and marble surfaces.

**Fig. S14** The variation of λ with load for the robots in Table 1.

**Fig. S15** Variation of CoT with load for tethered and untethered prototypes.

**Fig. S16** CoT comparison of the proposed robot with similar piezoelectric robot.

**Other Supplemental Materials for this manuscript include the following：**

**Movie S1** Configuration and working principle of the robot.

**Movie S2** Forward motions of the robot with different *Angle* (10°, 15°, 20°, 25°, 30°) and positions of weights (back, middle and front).

**Movie S3** Rotational motions of the robot with different *Angle* (10°, 15°, 20°, 25°) and positions of weights (back, middle and front).

**Movie S4** Forward motions of the robot under different loads.

**Movie S5** Flexibility testing of the robot.

**Movie S6** Motion characteristics testing of the untethered robot.

**Movie S7** Forward motions of the untethered robot under different loads.

**Movie S8** Demonstrative experiment 1: endurance capability testing of the untethered robot.

**Movie S9** Demonstrative experiment 2: navigating through a simulated narrow environment.

**Movie S10** Comparison of the previous and updated untethered prototypes.

**Note S1 Deformation principle of the driving leg.**

As shown in Fig. S2(A), the PZT elements are a sandwich structure composed of silver layers on the upper and lower surfaces and PZT material in the middle. The silver layers enable the electric field to be uniformly applied to the surface of the PZT elements. According to the inverse piezoelectric effect, when the applied electric field is opposite to the polarization direction of the PZT elements, the elements elongate. When the directions are the same, the PZT elements contract. The deformation principle of the driving leg is illustrated in Fig. S2(B). PZT elements are bonded to the four surfaces of the base beam using epoxy resin, with the wiring method shown in Fig. 2(A). When a sinusoidal signal is applied to the PZT elements, the deformation direction of the PZT elements on the opposite side of the base beam is always opposite. This opposite deformation enables the driving leg to achieve bending deformation.

**Note S2 The method for achieving mode degeneration in the ZOX and YOX planes.**

The most direct approach to bringing the resonant frequencies of the first order bending modes in the ZOX and YOX planes as close as possible (mode degeneration) is to ensure that the driving leg appears structurally identical when viewed from the two directions. However, to facilitate mounting of the driving leg, the connecting beam must be designed along the Y direction. To minimize its impact on the resonant frequencies in both directions, the connection should be positioned near the node of the first order bending mode (where vibration amplitude is zero). Additionally, the thickness of the connecting beam should be minimized while still ensuring sufficient structural rigidity. Following the above design principles to optimize structural parameters enables mode degeneration of the driving leg in simulation. During fabrication, the main structural dimensions of the driving leg also require tighter manufacturing tolerances (±0.02 mm).

**Note S3: Analysis of the tethered robot load characteristic testing results.**

Analysis of the first point is as follows: the simplified force model of the robot at rest under load is shown in Fig. S9. The combined weight of the robot and load is represented by the equivalent gravity *G*, while the ground support forces on the foot and passive wheels are denoted as *F*1 and *F*2, respectively. Force and moment balances of the above model yield:

Solving gives , .

When the load is positioned closer to the front, the application point of the equivalent gravity *G* shifts toward the foot, decreasing *l*1 and increasing *l*2. Consequently, *F*1 increases while *F*2 decreases. When the load mass increases, the magnitude of the equivalent gravity *G* increases, causing both *F*1 and *F*2 to increase. Friction force is positively correlated with normal pressure. As *F*1 increases, the ground friction on the foot (the driving force for forward motion) also increases during robot motion, reducing relative slip between the foot and the ground.

The second point stems from empirical patterns during experimentation. The primary resistance during forward motion for our robot originates from the rolling resistance of the passive wheels (bearings). Compared with the sliding friction between the foot and the ground (the driving force), the rolling resistance of the bearings increases to a lesser extent with added load mass. We therefore conclude that increasing load mass has a relatively minor effect on the motion resistance of our robot with the driving leg and passive wheels support scheme.

**Note S4: Detailed calculation process of the CoT of our robot.**

CoT could be calculated by Equation (2):

where *P* represents the power consumption (unit: W), *m* and *v* represent the mass (unit: kg) and the velocity (unit: m/s) of the robot, *g* represents the gravitational acceleration (with a value of 9.8 m/s2). Fig. 4I and 4J illustrate that with an excitation signal of 120 Vp-p and a load of 200 g (total weight 208.6 g), our tethered robot achieves a forward velocity of 313.49 mm/s and a power consumption of 2.30 W. As derived from Equation (2), the CoT value is 3.59.

As shown in Fig. 5H, our untethered robot achieves a forward velocity of 265.37 mm/s under a voltage of 80 Vp-p with a load of 180 g (total weight 200.86 g). As shown in Fig. 4J, the power consumption is 1.00 W when the exciting voltage is 80 Vp-p. As derived from Equation (2), the CoT value of the untethered robot is only 1.91. It should be noted that the power consumption of piezoelectric robots depends solely on the excitation voltage and shows no significant relationship with load conditions. Therefore, the power data used for CoT calculation can be directly taken from the results shown in Fig. 4J (measured under no load conditions).

**Supplemental Figures**

**
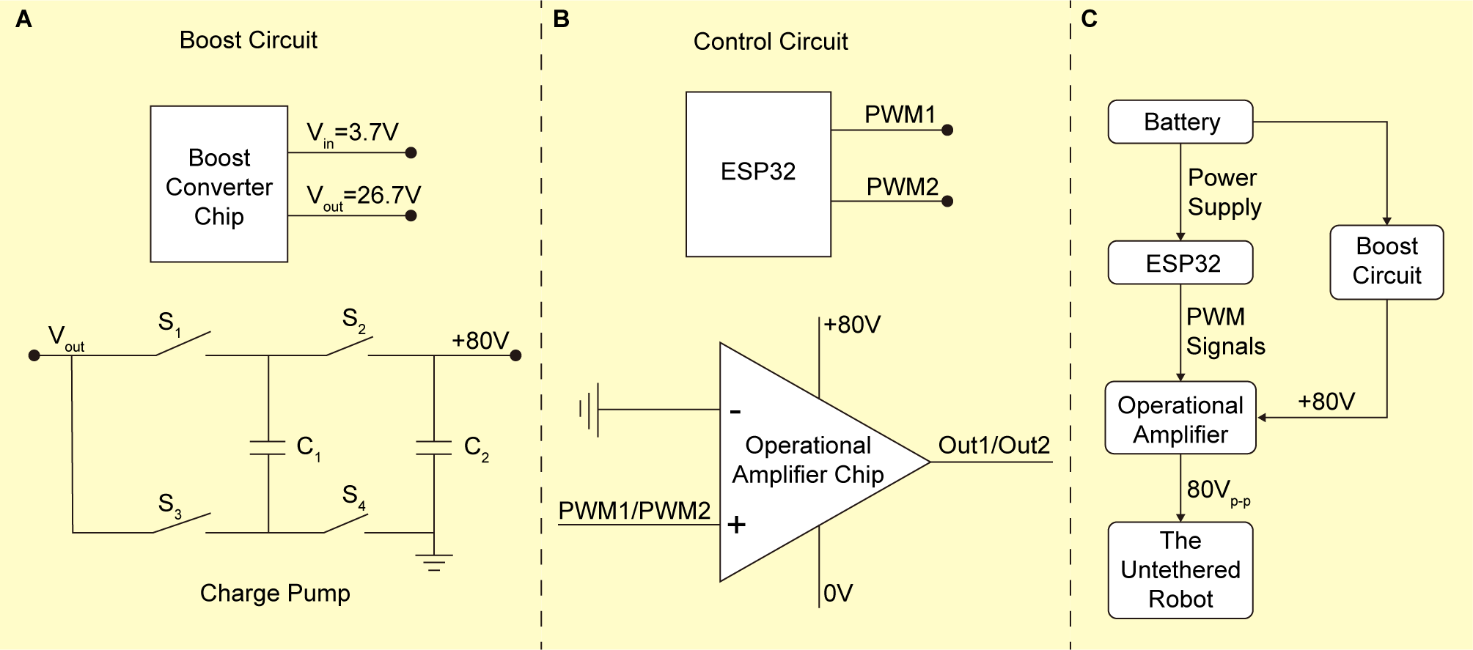
**

Fig. S1. Circuit schematic diagram of the control unit. (A) Circuit schematic diagram of the boost circuit. (B) Circuit schematic diagram of the control circuit. (C) Relationship among the parts of the control unit.


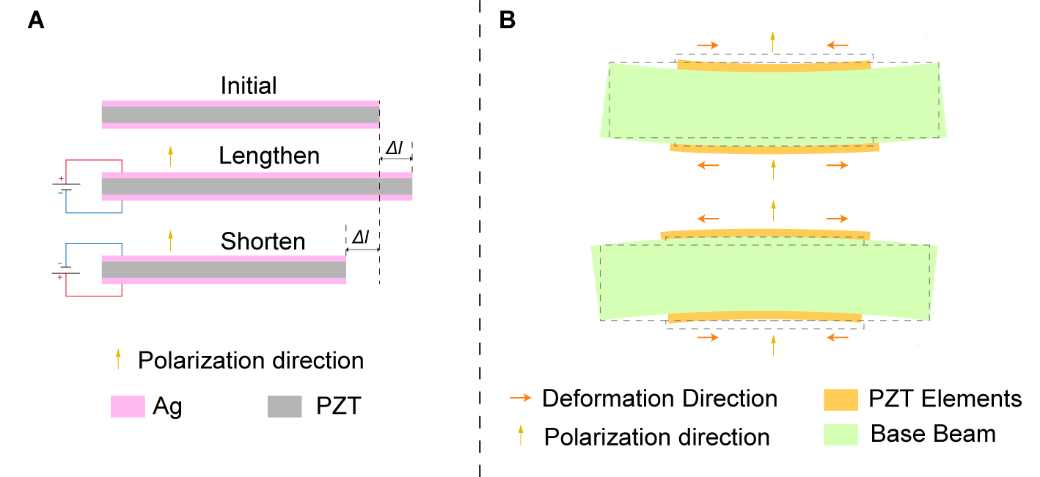


Fig. S2. Deformation principle of the driving leg. (A) Deformation of PZT elements under the action of electric field. (B) Deformation of the driving leg.


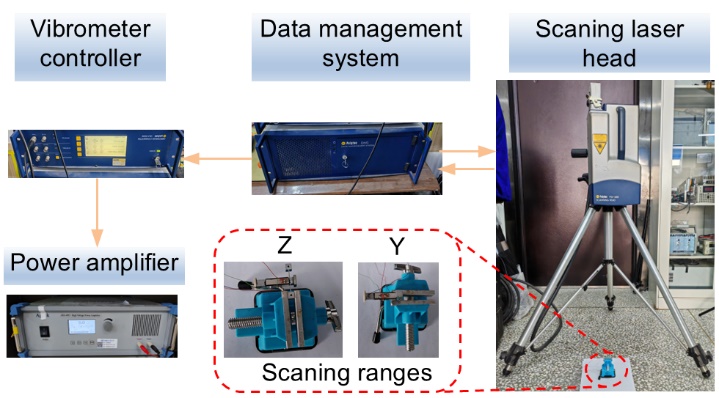


Fig. S3. Experimental setups for vibration modes testing of the driving leg.


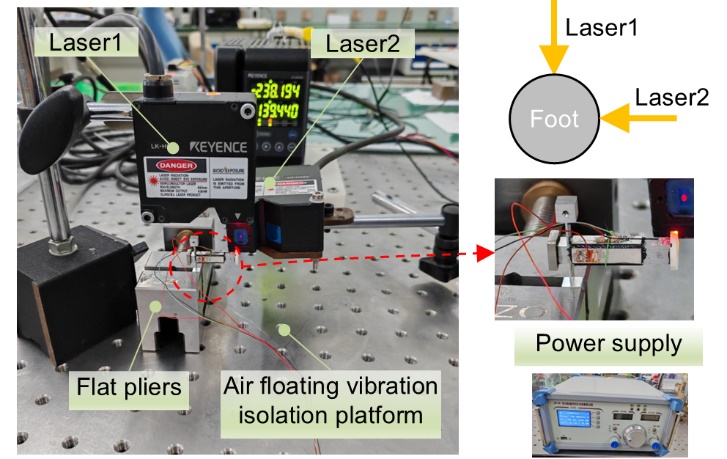


Fig. S4. Experimental setups for testing the displacements and trajectories of the foot.

**
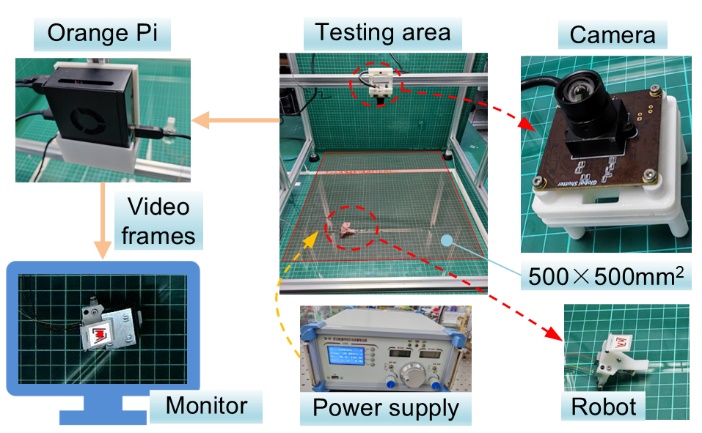
**

Fig. S5. Experimental setups for testing motion characteristics of the robot.


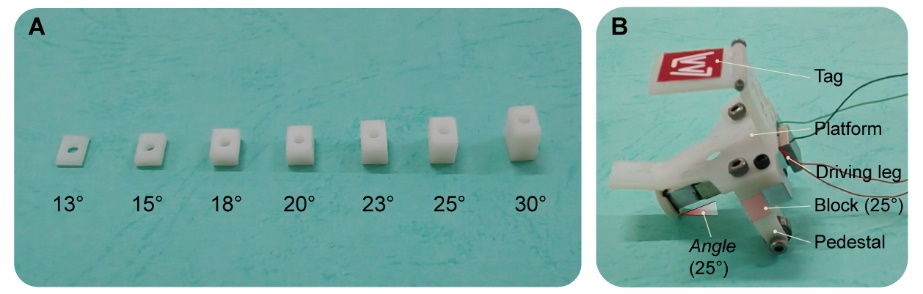


Fig. S6 Method for adjusting the angle between the driving leg and the ground. (A) Blocks of different thicknesses. (B) Position of the block on the supporting leg.


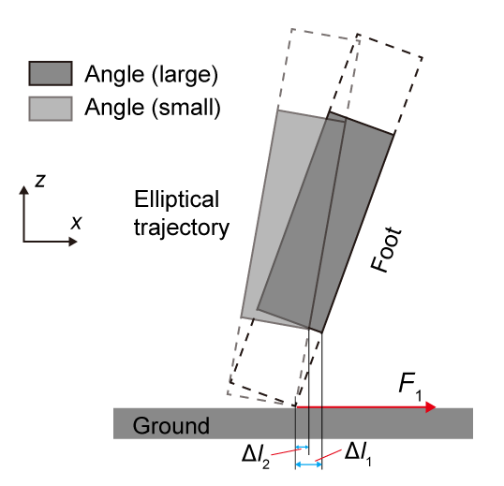


Fig. S7. Influence of the *Angle* variation on the projection component of foot vibration trajectory in the positive X direction.


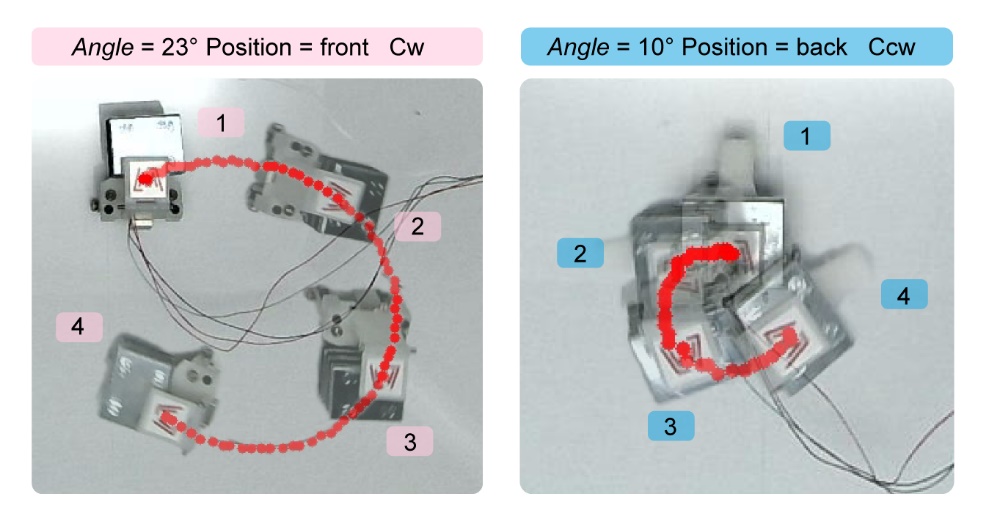


Fig. S8. Display of the rotational motion processes of the robot under different conditions


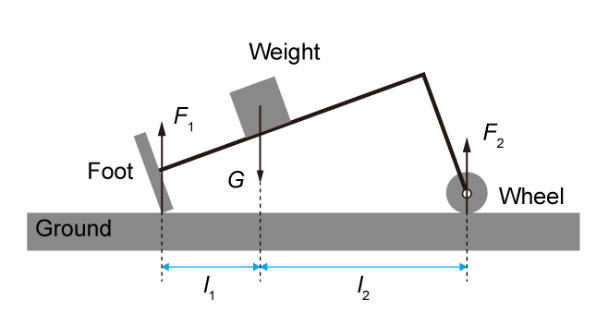


Fig. S9. Simplified static force model of the robot under load.


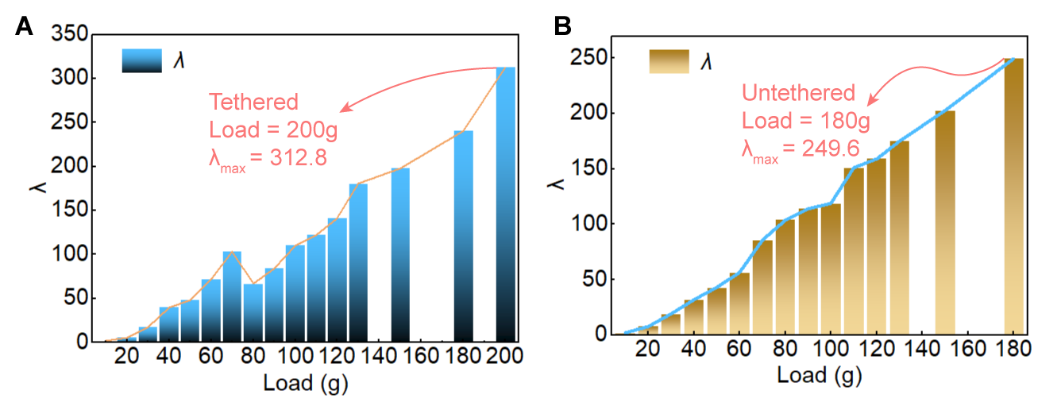


Fig. S10. Variation of the λ values with load mass for both tethered and untethered robots. (A) Variation of the λ values with load mass for tethered robot. (B) Variation of the λ values with load mass for untethered robot.


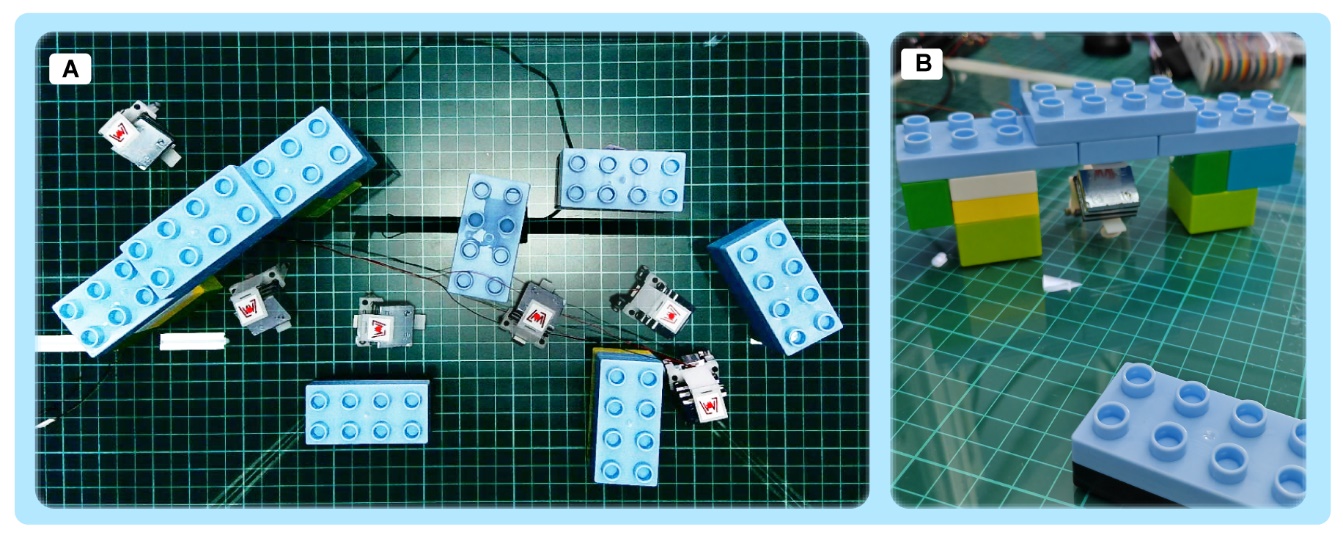


Fig. S11. Flexibility testing of the tethered robot. (A) Display of the robot’s motion process in the flexibility testing. (B) Display of the robot through the narrow tunnel.


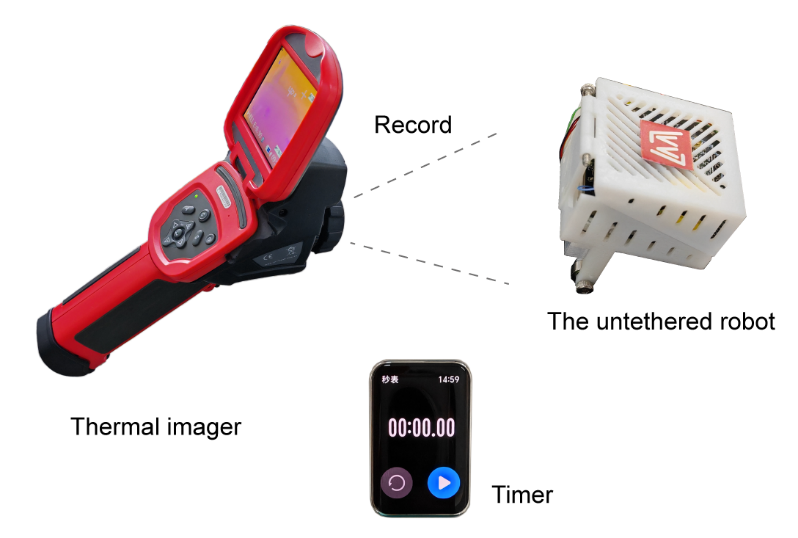


Fig. S12. Experimental setups for measuring the temperature of the untethered robot.


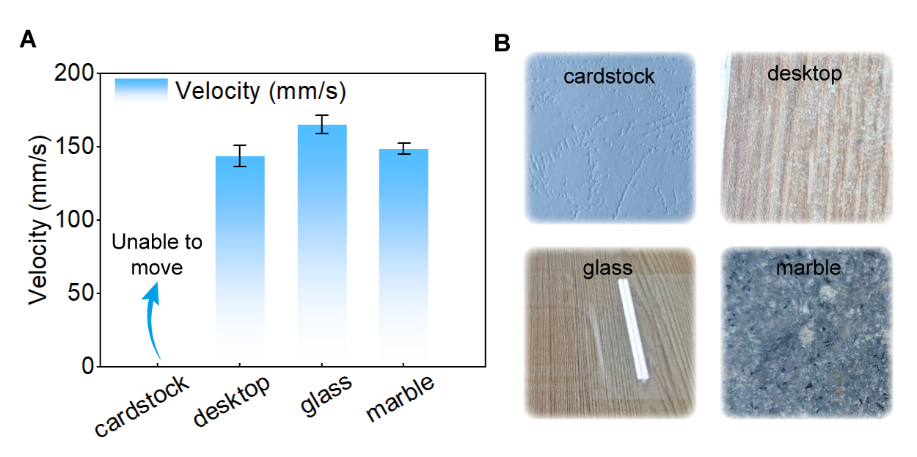


Fig. S13. Motion experiments of the robot on cardstock, desktop, glass, and marble surfaces. (A) Velocity of the robot on different surfaces. (B) Photographs of different surfaces.


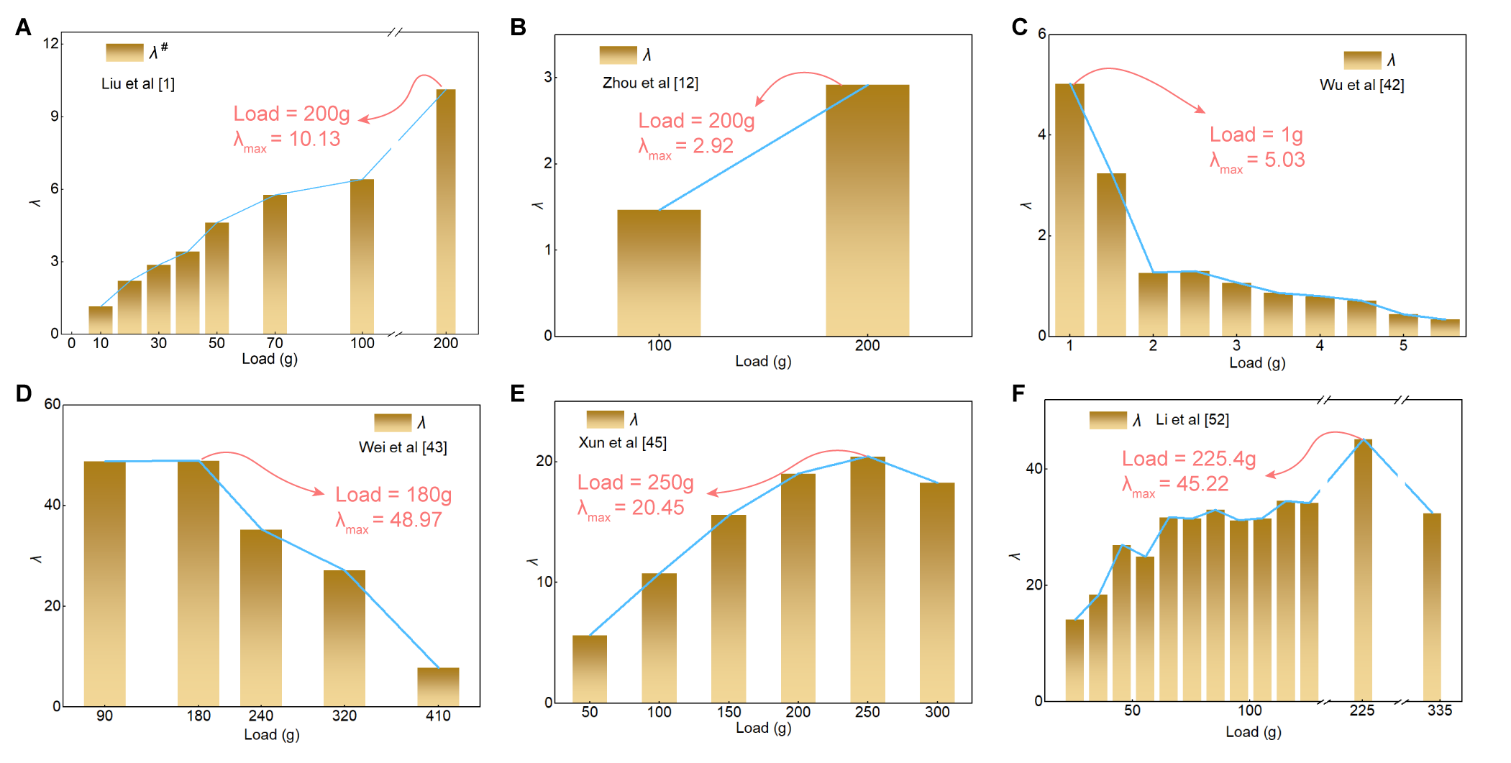


Fig. S14. The variation of *λ* with load for the robots in Table 1. (A) Variation of *λ* with load for Liu et al.’s robot. (B) Variation of *λ* with load for Zhou et al.’s robot. (C) Variation of *λ* with load for Wu et al.’s robot. (D) Variation of *λ* with load for Wei et al.’s robot. (E) Variation of *λ* with load for Xun et al.’s robot. (F) Variation of *λ* with load for Li et al.’s robot. # The *λ* value for each load is calculated based on the maximum velocity recorded at the current load.


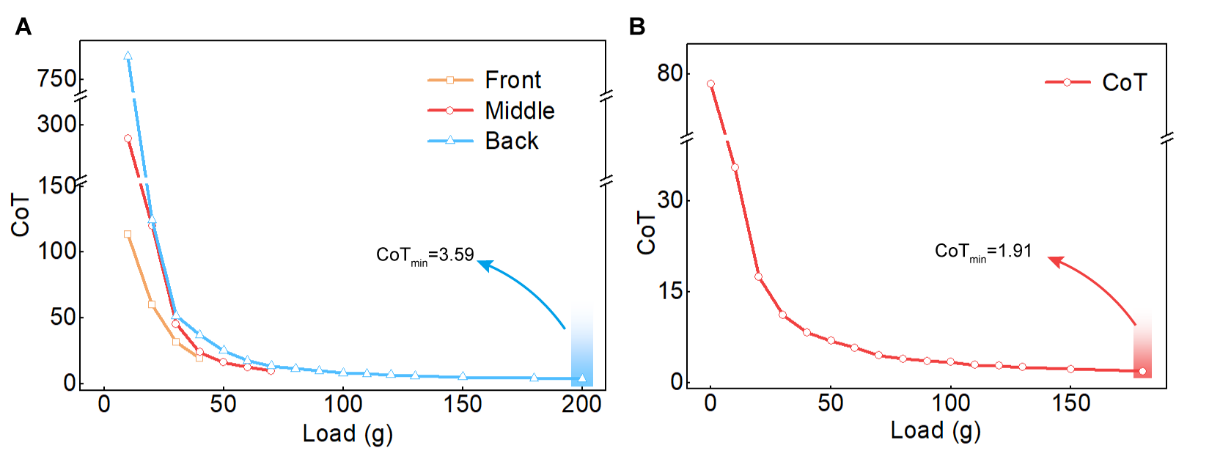


Fig. S15. Variation of CoT with load for tethered and untethered prototypes. (A) Variation of CoT with load for the tethered prototype. (B) Variation of CoT with load for the untethered prototype.


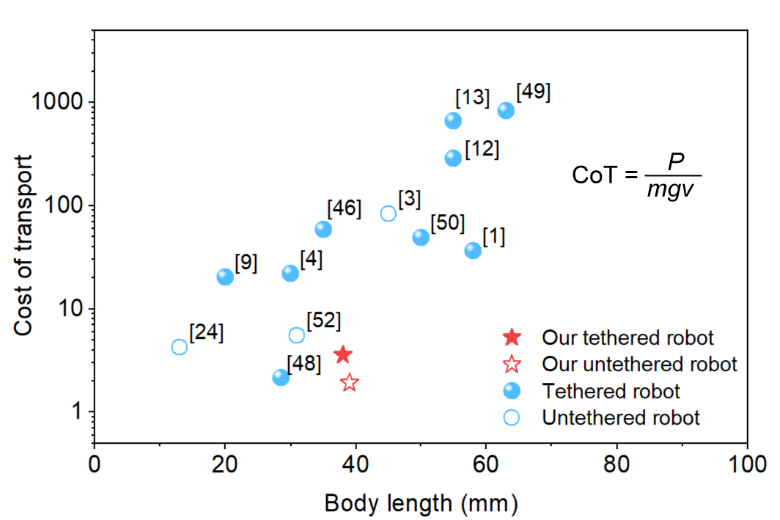


Fig. S16. CoT comparison of the proposed robot with similar piezoelectric robot. Part of the CoT data is not directly provided and is calculated from the known data in the paper. The numbers near the symbols identify the source of the reference.
